# The relationships between distances covered above generic and relative speed thresholds by male soccer players in English Premier League matches across two competitive seasons. The effects of positional demands and possession

**DOI:** 10.5114/biolsport.2024.135416

**Published:** 2024-03-18

**Authors:** Ronan Kavanagh, Rocco Di Michele, Rafael Oliveira, Kevin McDaid, David Rhodes, Ryland Morgans

**Affiliations:** 1 Nottingham Forest FC, Nottingham, UK; 2 Department of Biomedical and Neuromotor Sciences, University of Bologna, Italy; 3 Sports Science School of Rio Maior-Polytechnic Institute of Santarém, 2040-413 Rio Maior, Portugal; 4 Research Centre in Sport Sciences, Health Sciences and Human Development, 5001-801 Vila Real, Portugal; 5 Life Quality Research Centre, 2040-413 Rio Maior, Portugal; 6 Dundalk Institute of Technology, Dundalk, Co Louth, Ireland; 7 Human Performance Department, Burnley Football Club, Burnley; 8 School of Sport and Health Sciences, Cardiff Metropolitan University, Cardiff

**Keywords:** Match performance, Individualized speed zones, Performance analysis, Soccer, External load, External intensity

## Abstract

The aims of this study were to: a) examine the relationships between high-intensity distances covered above generic and relative speed thresholds in English Premier League (EPL) matches across two consecutive seasons and b) analyze the effects of playing position and team possession. Sixteen elite male soccer players (seven defenders, six midfielders and three forwards) participated in this study (age 27.8 ± 3.5 years, height 183.7 ± 5.4 cm, body mass 83.9 ± 7.1 kg). An Optical Tracking System was used to collect the following variables: total distance covered; high-speed running distance (HSRD) (> 5.5 m/s); high-intensity running distance (HIRD) (5.5–7 m/s); sprint distance (> 7 m/s); total distance covered above Maximal Aerobic Speed (MAS); distance covered > 85% peak speed (PS); and distance > 30% Anaerobic Speed Reserve (ASR). All measures were analyzed as whole match totals and as distances covered in the periods of the team in possession (TIP), opponent team in possession (OTIP), and ball out of play (BOP). Analysis by position based on defenders, midfielders and forwards was also performed. Distance > 30% ASR was almost perfectly correlated with HSRD (r = 0.98), while distances > MAS were highly correlated with both HIRD (r = 0.91) and HSRD (r = 0.91), and distance > 85% PS were highly correlated with SD (r = 0.70). Although the generic and relative speed thresholds show almost perfect correlation, the differences between HSRD, HIRD and distance > MAS indicate that players may be exposed to more HIRD when using relative thresholds.

## INTRODUCTION

Soccer is an intermittent field sport containing both high- and lowintensity running efforts [1]. The monitoring of physical performance in elite soccer has developed in recent years with advancements in suitable player tracking technologies [2]. Accurately quantifying players’ match actions is required to improve the understanding of workloads during match-play [3]. When match-play physical demands are examined, load is often characterized by graduated speed thresholds ranging from motionless standing to maximal sprinting [4]. Player tracking has traditionally been reported using generic speed zones [5]. Currently, there is no consensus on the metrics that are most practically useful, specific and reliable [6]. For instance, a recent systematic review noted that there is a lack of uniformity to classify different speed thresholds which makes comparisons between studies or generalizations of results difficult [7]. Historically, generic speed thresholds have been applied to all squad athletes [8]. This allows the comparison of physical performance between players within and across teams and leagues to be conducted. However, these thresholds do not account for individual physical differences and the relative exertion imposed on the player to reach such generic speeds.

The disadvantages associated with generic speed thresholds have been documented, thus, researchers have attempted to individualize thresholds using physical performance markers [5, 9–11]. The aim of individualizing speed thresholds is to account for the individual nature of the exercise-intensity continuum and accurately represent the relative intensity of an athlete when performing [8]. Varying methodological approaches employed to individualize high-speed thresholds, such as anaerobic threshold, have been reported, however, this can be difficult to implement in team environments with large squads [12]. Thus, another marker, namely percentage of peak speed (PS) has been utilized to underpin high-intensity running distance (HIRD) in team athletes [13]. However, Hunter, et al. [5] found that utilizing a single physical marker to determine multiple speed zones can lead to erroneous interpretations of players activities. Additionally, it has been suggested that a measure that describes the functional limits of endurance and an additional value that characterizes sprint capacity may be ideal [14].

Maximal Aerobic Speed (MAS) has been defined as a practical and time efficient method to assess the aerobic energy system in team sport athletes [12]. Time spent above MAS has been shown to correlate with improvements in aerobic fitness [14]. Recently several authors applied this to youth athletes, using field tests to assess MAS and PS [5, 10, 15]. This approach also allows for the estimation of an individual’s Anaerobic Speed Reserve (ASR) and transition to sprint distances [5]. Therefore, an individualized approach to external load monitoring may also augment practitioners understanding of competition demands [5, 16]. The application of generic thresholds may also lead to an inaccurate external load quantification by sports science practitioners [17], whereas individualized thresholds have been proposed as a method to overcome this weakness [18]. Despite this rationale, individualized speed thresholds are not widely accepted in high-level soccer [19].

To date, no study has examined the differences in HIRD covered above generic and relative speed thresholds in English Premier League (EPL) matches. Examining league data is potentially key to improving the understanding of various methods of physical development [20]. These physical demands can also differ significantly depending on playing position and possession [21–25]. While in possession, players perform multiple high-speed and sprint activities in an attempt to create chances and score goals [26, 27]. Similarly, when not in possession, players still produce high-speed and sprint actions in an attempt to recover possession [26]. Indeed elite UEFA Champions League players produce greater efforts while not in possession across all positions [28]. It is therefore important to fully understand the differences in HIRD covered above generic and relative speed thresholds in and out of possession during match-play, to provide practitioners with detailed information on player exertion in order to design and deliver individually tailored sessions and weekly loads based on scientific principles. Further research is therefore warranted on the positional demands and effects of team possession on distance > MAS, ASR and distances > 85% PS.

Thus, the primary aim of this study was to examine the relationships between different generic and relative speed thresholds in EPL matches across two competitive seasons (2019–20 and 2020–2021). The secondary aim was to investigate the effect of playing position and team possession. The study hypothesis was that playing position and team possession will influence the quantity of distances covered above generic and relative speed thresholds in EPL match-play.

## MATERIALS AND METHODS

### Participants

Sixteen male professional outfield soccer players (mean ± standard deviation (SD), at the start of 2019–2020 season, age 27.8 ± 3.5 years, height 183.7 ± 5.4 cm, body mass 83.9 ± 7.1 kg) from an EPL team participated in the present study. The methodology to differentiate specialized positions was adapted from previous research [29] as various situational factors have an influence on the style of play that can be modulated by different tactical roles [30]. The small sample size is supported by previous studies in elite soccer cohorts [31–33], and consisted of three main positions, namely, defenders n = 7, midfielders n = 6, and forwards n = 3. Goal-keepers data were excluded due to their position-specific demands [34, 35]. Players’ positional data were collected from 38 matches across two consecutive seasons (2019–2020 and 2020–2021). All data evolved as a result of employment where players were routinely monitored over the course of the competitive season [36]. Nevertheless, club approval for the study was obtained [37] and ethics was provided by the local Ethics Committee of University of Central Lancashire (BAHSS 646 dated 17/04/2019) and in accordance with the Helsinki Declaration. Moreover, all players provided written consent to participate in the study. To ensure confidentiality, all data were anonymized before analysis.

### Procedure

In each season, only data from 19 home league matches and 19 away league matches from the EPL were included in the analysis. Participant data were only included in the analyses when time spent on the field exceeded 75-minutes of the match [38]. For each season, players were considered when an inclusion criterion of 75-minutes playing time, in eight (10.5%) or more league matches across the two-season examined period was fulfilled. Only players with match data from both examined seasons were included in the sample. The participants performed in a median of 60% (range = 20 to 97%) of league matches across both seasons. A total of 630 individual match data points were examined across both seasons, with a median of 40.5 matches per player (range = 8 to 74). No data from international camps (training or matches) was included.

### Data collection

League match data across both study seasons was recorded and analyzed via an Optical Tracking System (Second Spectrum®, Los Angeles, USA). Second spectrum optical tracking has recently been detailed by the FIFA program to meet industry standards [39]. Data was collected via semi-automated HD cameras that were positioned around the stadium at a sampling frequency of 25-Hz. The Second Spectrum match data was processed directly using the python programming language (Python 2.7) through the Spyder scientific development environment (https://www.spyder-ide.org/).

The metrics used for analysis across each season were: total distance covered; high-speed running distance (HSRD) (> 5.5 m/s); high-intensity running distance (HIRD) (5.5–7 m/s); sprint distance (> 7 m/s) [19]; total distance covered > MAS (MAS) [5, 14]; distance covered > 85% PS (PS) [40]; and distance > 30% ASR (ASR) [8]. All distances were examined as whole match totals and as distances covered in the periods of team in possession (TIP), opponent team in possession (OTIP), and ball out of play (BOP). All examined metrics were expressed both in meters and m/min (whole match, TIP, OTIP and BOP distances were respectively divided by the total, TIP, OTIP and BOP times). Before calculating these values, when individual match playing time was less than 90-minutes, distances were extrapolated to 90-minutes utilizing the meters per minute calculation. All variables obtained were calculated or pre-determined in the Second Spectrum System Software. These variables have been previously utilized by soccer practitioners to longitudinally track the external load undertaken by players [19]. The installation process, reliability and validity of Second Spectrum have been reported recently by FIFA Electronic Performance Tracking Systems (EPTS) programme [39].

### Maximal aerobic speed test (MAS)

**FIG. 1:**
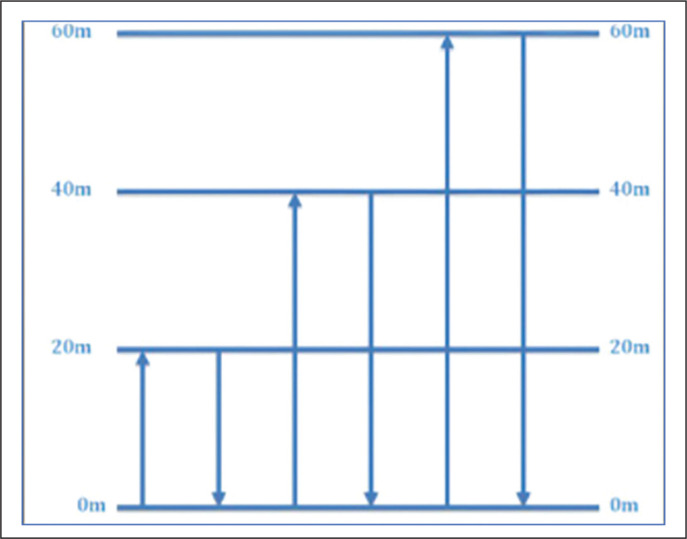
Shows the Bronco 1200m shuttle test

During the pre-season period, participants completed a MAS test to estimate velocity at the maximum oxygen consumption (vVO^2^max). The MAS protocol was a 1200 m maximum effort shuttle test. The 1200 m shuttle test has previously shown a strong correlation with other MAS tests [12, 41]. Poles were set at the start point, and 20 m, 40 m and 60 m from the starting point (see Figure 1). Players were instructed to run from the start point to 20 m and return to the start point. Players then ran to the 40 m mark and returned to the start point before running to the 60 m mark and returning to the start point. This sequence was repeated as quickly as possible five times until the distance of 1200 m had been achieved [41]. Players were informed of how much time was remaining at 1-minute intervals until the test was complete to ensure players were performing maximally [42]. This verbal encouragement has been shown to be a motivational requirement for laboratory assessments of time to exhaustion and central fatigue [43]. Due to the change of direction within the test, a corrective equation was employed, 1200/(Time – 20.3 s (0.7 s for each turn) = MAS (m/s) [12].

### Maximal peak speed (PS)

Each player’s maximum PS during the season was collated using Optical Tracking System (Second Spectrum®, Los Angeles, USA). The researchers decided to use the individual maximum PS from match-play only, as an average PS per session may be influenced by positional demands and therefore would not be a true reflection of the players PS capacity. If a player produced a new PS during the season this was adjusted within the python programming language (Python 2.7), using the Spyder scientific development environment (https://www.spyder-ide.org/).

### Anaerobic speed reserve (ASR)

Using MAS and PS scores, each athlete’s theoretical ASR was calculated. ASR was defined as the difference between the MAS and PS score and reported in m ∙ s^−1^. The MAS and PS protocols were previously utilised by Kavanagh et al. [36] to determine a soccer player’s MAS and MSS. The 30% ASR measure employed a weighted MAS value and the MSS for each player using 70% and 30% respectively as previous reported [36].

### Statistical analysis

The analyses were carried using the software R, version 4.2.0 (R Foundation for Statistical Computing, Vienna, Austria), with packages lme4 and rmcorr. All variables are shown as the mean ± SD. Repeated-measure correlations were calculated to examine the relationships between the examined physical performance variables, including distances covered between or above the selected generic and individualized speed thresholds. This technique allows the calculation of paired correlations using data obtained from repeated measures on multiple individuals as in the present data set, without violating the assumption of data independence. The repeated-measure correlation (rmcorr) represents the strength of the linear association between two variables, assessed as the common individual association [44]. The magnitude of rmcorr was interpreted as Pearson’s r correlation coefficient as trivial (< 0.1), small (0.1–0.3), moderate (0.3–0.5), large (0.5–0.7), very large (0.7–0.9), and almost perfect (> 0.9) [45].

Linear mixed models with random intercept for individual players were used to compare the examined physical performance variables, standardized by time on pitch, across playing positions (defender, midfielder, forward), separately for each playing period (whole match, TIP, OTIP, BOP). When there was a significant (p < 0.05) effect for playing position, Tukey’s tests were used to examine which positions differed. The differences were standardized by the betweensubject standard deviation to determine the effect size (ES), and were evaluated as < 0.2, trivial; 0.2–0.6, small; 0.6–1.2, moderate; 1.2–2.0, large; 2.0–4.0, very large; > 4.0 extremely large [45].

## RESULTS

The analysis of the results in Table 1 allowed us to conclude that for all the variables there were no grounds to reject the null hypothesis of normality of the variables analyzed, so in subsequent analyses the statistics were based on parametric tools, namely analysis of variance with repeated measures and Tuckey’s post hoc multiple comparison tests. Analysis of the results for the 5 m [s] distance (Fig. 1) revealed significant differences for the main effects: differences between groups (F = 6.61; p = 0.015; ɳ^2^ = 0.18); differences for the main effects before and after (F = 93.89; p < 0.0001; ɳ^2^ = 0.75); and for the interaction group × measurement time (F = 15.85; p = 0.0004; ɳ^2^ = 0.35). To determine between which groups there were significant differences for the interactions, Tuckey’s post hoc multiple comparison tests were used. No significant differences were found between the results of the 5 m [s] test in the groups analyzed before the experiment after the experiment (p = 0.0023; d = 1.57). After the experiment, the time needed to run the distance was statistically significantly shorter in the experimental group than in the control (Fig. 1).

**TABLE 1:** Mean ± *SD* values for MAS, PS, and ASR

	All players (n = 16)	Defenders (n = 7)	Midfielders (n = 6)	Forwards (n = 3)
MAS (m/s)	4.63 ± 0.19	4.62 ± 0.19	4.75 ± 0.12	4.51 ± 0.25
PS (m/s)	9.53 ± 0.39	9.63 ± 0.35	9.37 ± 0.43	9.83 ± 0.32
ASR (m/s)	4.89 ± 0.45	5.01 ± 0.24	4.61 ± 0.50	5.32 ± 0.47

Table 1 shows the mean MAS, PS, and ASR in the present sample of players.

The average whole match playing time for the individual match data points was 95.5 ± 4.1-minutes, whereas TIP, OTIP and BOP times were 21.9 ± 3.6-minutes (22.9%), 30.6 ± 5.7-minutes (32%), and 43.1 ± 5.9-minutes (45.1%) respectively.

For descriptive purposes, the mean ± SD values of all examined physical performance variables are reported in Table 2.

**TABLE 2:** Mean ± *SD* values for the examined distance metrics

Variables	Positions	Whole match	TIP	OTIP	BOP
**Total distance (m)**	Defenders	9966 ± 515	2748 ± 504	4477 ± 713	2740 ± 405
	Midfielders	11594 ± 622	3531 ± 604	5089 ± 926	2974 ± 420
	Forwards	10710 ± 573	3579 ± 501	4303 ± 823	2829 ± 444
	Overall	10668 ± 945	3140 ± 673	4689 ± 871	2839 ± 429

**High-speed running distance (> 5.5 m/s) (m)**	Defenders	697 ± 275	182 ± 159	478 ± 157	37 ± 36
	Midfielders	992 ± 205	331 ± 145	627 ± 166	34 ± 31
	Forwards	941 ± 182	535 ± 130	363 ± 133	44 ± 30
	Overall	837 ± 280	279 ± 189	521 ± 181	37 ± 34

**High-intensity running distance (5.5–7 m/s) (m)**	Defenders	546 ± 195	135 ± 104	380 ± 121	31 ± 28
	Midfielders	834 ± 160	277 ± 111	526 ± 140	31 ± 26
	Forwards	740 ± 137	400 ± 86	303 ± 111	36 ± 24
	Overall	677 ± 223	219 ± 140	427 ± 151	32 ± 27

**Sprint distance (> 7 m/s) (m)**	Defenders	151 ± 95	47 ± 62	98 ± 55	6 ± 13
	Midfielders	158 ± 80	54 ± 50	101 ± 52	3 ± 8
	Forwards	202 ± 84	135 ± 61	60 ± 42	7 ± 11
	Overall	160 ± 89	60 ± 64	95 ± 54	5 ± 11

**Distance > MAS (m)**	Defenders	1414 ± 412	421 ± 244	896 ± 262	96 ± 60
	Midfielders	1868 ± 292	687 ± 202	1103 ± 270	78 ± 52
	Forwards	2010 ± 241	1110 ± 187	791 ± 219	110 ± 55
	Overall	1654 ± 431	601 ± 314	962 ± 284	91 ± 57

**Distance > 30% ASR (m)**	Defenders	589 ± 217	166 ± 133	393 ± 145	30 ± 30
	Midfielders	726 ± 163	250 ± 109	453 ± 142	23 ± 25
	Forwards	765 ± 150	460 ± 114	270 ± 102	35 ± 27
	Overall	661 ± 204	232 ± 152	401 ± 150	28 ± 28

**Distance > 85% PS (m)**	Defenders	24 ± 24	7 ± 13	16 ± 20	1 ± 4
	Midfielders	31 ± 27	10 ± 16	21 ± 20	0 ± 2
	Forwards	20 ± 18	16 ± 16	3 ± 7	1 ± 3
	Overall	26 ± 25	9 ± 15	16 ± 20	1 ± 3

All metrics represent means and standard deviations for each position.

The matrix of repeated-measure correlations with 95% confidence intervals for the examined variables is shown in Table 3.

**TABLE 3:** Repeated-measure correlations (with 95% CIs) between the examined distance metrics.

	Total distance	High-speed running distance (m)	High-intensity running distance (m)	Sprint distance (m)	Distance > MAS (m)	Distance > 30% ASR (m)
**High-speed running distance**	ALL: 0.41^*^ (0.34–0.48)					
	
	TIP: 0.49^*^ (0.43–0.55)					
	
	OTIP: 0.47^*^ (0.41–0.53)					
	
	BOP: 0.40^*^ (0.34–0.47)					
	
**High-intensity running distance**	ALL: 0.45^*^ (0.39–0.51)	ALL: 0.93^*^ (0.92–0.94)				
	
	TIP: 0.55^*^ (0.49–0.60)	TIP: 0.94^*^ (0.93–0.95)				
	
	OTIP: 0.51^*^ (0.45–0.57)	OTIP: 0.95^*^ (0.94–0.96)				
	
	BOP: 0.41^*^ (0.34–0.47)	BOP: 0.95^*^ (0.94–0.96)				
	
**Sprint distance**	ALL: 0.16^*^ (0.08–0.24)	ALL: 0.72^*^ (0.68–0.75)	ALL: 0.42^*^ (0.36–0.49)			
	
	TIP: 0.23^*^ (0.15–0.30)	TIP: 0.77^*^ (0.73–0.80)	TIP: 0.49^*^ (0.43–0.55)			
	
	OTIP: 0.17^*^ (0.09–0.24)	OTIP: 0.68^*^ (0.64–0.72)	OTIP: 0.41^*^ (0.35–0.48)			
	
	BOP: 0.24^*^ (0.16–0.31)	BOP: 0.72^*^ (0.67–0.75)	BOP: 0.46^*^ (0.39–0.52)			
	
**Distance > MAS**	ALL: 0.58^*^ (0.52–0.63)	ALL: 0.91^*^ (0.90–0.92)	ALL: 0.91^*^ (0.90–0.92)	ALL: 0.54^*^ (0.48–0.59)		
	
	TIP: 0.57^*^ (0.52–0.63)	TIP: 0.86^*^ (0.84–0.88)	TIP: 0.86^*^ (0.83–0.88)	TIP: 0.58^*^ (0.52–0.63)		
	
	OTIP: 0.61^*^ (0.56–0.66)	OTIP: 0.88^*^ (0.86–0.90)	OTIP: 0.88^*^ (0.86–0.90)	OTIP: 0.50^*^ (0.43–0.55)		
	
	BOP: 0.50^*^ (0.44–0.56)	BOP: 0.90^*^ (0.89–0.91)	BOP: 0.88^*^ (0.86–0.89)	BOP: 0.58^*^ (0.52–0.63)		
	
**Distance > 30% ASR**	ALL: 0.36^*^ (0.29–0.43)	ALL: 0.98^*^ (0.98–0.98)	ALL: 0.89^*^ (0.87–0.90)	ALL: 0.76^*^ (0.73–0.80)	ALL: 0.86^*^ (0.84–0.88)	
	
	TIP: 0.35^*^ (0.28–0.42)	TIP: 0.93^*^ (0.92–0.94)	TIP: 0.82^*^ (0.80–0.85)	TIP: 0.80^*^ (0.77–0.83)	TIP: 0.86^*^ (0.83–0.88)	
	
	OTIP: 0.41^*^ (0.34–0.47)	OTIP: 0.96^*^ (0.95–0.96)	OTIP: 0.88^*^ (0.86–0.90)	OTIP: 0.71^*^ (0.67–0.75)	OTIP: 0.88^*^ (0.86–0.89)	
	
	BOP: 0.39^*^ (0.32–0.45)	BOP: 0.98^*^ (0.97–0.98)	BOP: 0.90^*^ (0.88–0.91)	BOP: 0.76^*^ (0.72–0.79)	BOP: 0.86^*^ (0.83–0.88)	

**Distance > 85% PS**	ALL: 0.03 (-0.05–0.10)	ALL: 0.42^*^ (0.36–0.49)	ALL: 0.19^*^ (0.11–0.26)	ALL: 0.70^*^ (0.67–0.74)	ALL: 0.30^*^ (0.23–0.37)	ALL: 0.46^*^ (0.40–0.52)

	TIP: 0.10^*^ (0.02–0.18)	TIP: 0.47^*^ (0.41–0.53)	TIP: 0.26^*^ (0.19–0.33)	TIP: 0.68^*^ (0.64–0.72)	TIP: 0.39^*^ (0.32–0.45)	TIP: 0.54^*^ (0.48–0.60)

	OTIP: 0.08 (-0.00–0.16)	OTIP: 0.38^*^ (0.31–0.44)	OTIP: 0.18^*^ (0.10–0.25)	OTIP: 0.68^*^ (0.63–0.72)	OTIP: 0.29^*^ (0.21–0.36)	OTIP: 0.42^*^ (0.36–0.49)

	BOP: 0.16^*^ (0.08–0.23)	BOP: 0.46^*^ (0.39–0.52)	BOP: 0.27^*^ (0.19–0.34)	BOP: 0.70^*^ (0.66–0.74)	BOP: 0.35^*^ (0.28–0.42)	BOP: 0.49^*^ (0.43–0.55)

ALL = Whole Match;

* = p < 0.05

All correlations were significant (p < 0.05) with exception of correlations between total distance and distance > 85% PS for the whole match and the non-possession period. For any given pair of variables, there were no relevant differences between correlations as calculated when considering the whole match or the periods of possession, non-possession or ball out of play. There were almost perfect correlations between HSRD and both HIRD (rmcorr = 0.93, p < 0.001) and distance > 30% ASR (rmcorr = 0.98, p < 0.001). Also, HSRD showed very large correlations with sprint distance (rmcorr = 0.72, p < 0.01) and distance > MAS (rmcorr = 0.91, p < 0.001). Very large correlations were observed between HIRD and distance > MAS (rmcorr = 0.91, p < 0.001) and distance > 30% ASR (rmcorr = 0.89, p < 0.001), as well as between distance > 30% ASR and sprint distance (rmcorr = 0.76, p < 0.001) and distance > MAS (rmcorr = 0.86, p < 0.001). Very large correlations were also evident between distance > 85% PS and sprint distance (rmcorr = 0.70, p < 0.001). All other correlations ranged from small to large.

Table 4 shows the mean ± SD values for all examined distances, divided by time on pitch, across playing positions in all the considered match periods.

**TABLE 4:** Mean ± *SD* values for the examined distance metrics standardized by time on pitch.

	Positions	Whole match	TIP	OTIP	BOP
**Total distance (m/min)**	Defenders	103.2 ± 5.7	124.8 ± 10.7	145.5 ± 8.3	62.8 ± 4.8
	Midfielders	120.3^*^ ± 6.9	158.5^*^ ± 9.9	165.8^*^ ± 12.0	68.4^*^ ± 4.6
	Forwards	111.7^°^# ± 7.1	160.7# ± 8.8	140.6^°^ ± 10.5	65.9 ± 5.9
	Overall	111.0 ± 10.1	142.9 ± 19.9	152.7 ± 14.7	65.4 ± 5.5

**High-speed running distance (> 5.5 m/s) (m/min)**	Defenders	7.2 ± 2.9	8.2 ± 6.9	15.8 ± 5.4	0.8 ± 0.7
	Midfielders	10.3^*^ ± 2.1	15.1 ± 6.5	20.7^*^ ± 5.3	0.8 ± 0.7
	Forwards	9.9 ± 2.0	24.7^°^# ± 7.1	12.2^°^ ± 4.3	1.0 ± 0.7
	Overall	8.8 ± 2.9	13.2 ± 8.8	17.2 ± 6.0	0.8 ± 0.7

**High-intensity running distance (5.5–7 m/s) (m/min)**	Defenders	5.7 ± 2.0	6.1 ± 4.5	12.5 ± 4.2	0.7 ± 0.6
	Midfielders	8.7^*^ ± 1.7	12.5^*^ ± 4.8	17.3^*^ ± 4.3	0.7 ± 0.6
	Forwards	7.8# ± 1.5	18.6^°^# ± 4.7	10.2^°^ ± 3.6	0.8 ± 0.5
	Overall	7.1 ± 2.3	10.3 ± 6.4	14.1 ± 5.0	0.7 ± 0.6

**Sprint (> 7 m/s) distance (m/min)**	Defenders	1.6 ± 1.0	2.1 ± 2.8	3.2 ± 1.8	0.1 ± 0.3
	Midfielders	1.7 ± 0.8	2.5 ± 2.4	3.4 ± 1.8	0.1 ± 0.2
	Forwards	2.1 ± 0.9	6.1^°^# ± 3.2	2.0 ± 1.4	0.2 ± 0.3
	Overall	1.7 ± 0.9	2.9 ± 3.0	3.1 ± 1.8	0.1 ± 0.3

**Distance < MAS (m/min)**	Defenders	14.6 ± 4.3	19.2 ± 10.7	29.5 ± 8.9	2.2 ± 1.2
	Midfielders	19.4^*^ ± 3.1	31.3^*^ ± 8.7	36.2^*^ ± 8.2	1.8 ± 1.1
	Forwards	20.9# ± 2.8	50.6^°^# ± 10.9	26.1^°^ ± 6.3	2.5^°^ ± 1.2
	Overall	17.4 ± 4.5	28.3 ± 14.6	31.6 ± 9.2	2.1 ± 1.2

**Distance < 30% ASR (m/min)**	Defenders	6.1 ± 2.3	7.6 ± 6.0	12.9 ± 4.9	0.7 ± 0.6
	Midfielders	7.8^*^ ± 1.8	11.5 ± 5.3	15.0 ± 4.8	0.5 ± 0.6
	Forwards	7.9 ± 1.6	20.9^°^# ± 6.6	9.0^°^# ± 3.3	0.8^°^ ± 0.6
	Overall	6.9 ± 2.1	11.0 ± 7.3	13.2 ± 5.0	0.6 ± 0.6

**Distance < 85% PS (m/min)**	Defenders	0.2 ± 0.2	0.3 ± 0.6	0.5 ± 0.7	0.0 ± 0.1
	Midfielders	0.3 ± 0.3	0.5 ± 0.8	0.7 ± 0.7	0.0 ± 0.1
	Forwards	0.2^°^ ± 0.2	0.7 ± 0.8	0.1^°^# ± 0.2	0.0 ± 0.1
	Overall	0.3 ± 0.3	0.4 ± 0.7	0.5 ± 0.7	0.0 ± 0.1

* denotes a significant difference for midfielders vs. defenders;

° denotes a significant difference for forwards vs. midfielders;

# denotes a significant difference for forwards vs. defenders

When examining the whole match, midfielders covered greater total distance (p = 0.037, ES = 0.89, moderate) and distance > 85% PS (p = 0.047, ES = 1.74, large) than forwards. Compared to defenders, midfielders covered greater total distance (p < 0.001, ES = 1.91, large), HSRD (p = 0.009, ES = 1.33, large), HIRD (p < 0.001, ES = 1.59, large), distance > MAS (p = 0.002, ES = 1.36, large), and distance > 30% ASR (p = 0.035, ES = 1.19, moderate). Furthermore, forwards covered greater total distance (p = 0.012, ES = 1.01, moderate), HIRD (p = 0.032, ES = 1.23, large), and distance > MAS (p = 0.003, ES = 1.65, large) than defenders.

When the team was in possession of the ball, forwards, compared to both defenders and midfielders, covered greater HSRD (p < 0.001, ES = 2.13, very large, vs. defenders; and p = 0.016, ES = 1.28, large vs. midfielders), HIRD (p < 0.001, ES = 2.17, very large, vs. defenders; and p = 0.030, ES = 1.07, moderate vs. midfielders), sprint distance (p = 0.030, ES = 1.80, large, vs. defenders; and p = 0.012, 1.37, large vs. midfielders), distance > MAS (p < 0.001, ES = 2.26, very large vs. defenders and p = 0.002, ES = 1.36, large vs. midfielders), and distance > 30% ASR (p < 0.001, ES = 2.14, very large, vs. defenders; and p = 0.005, ES = 1.47 large, vs. midfielders). Also, forwards covered greater total distance than defenders (p < 0.001, ES = 1.90, large) and midfielders covered greater total distance (p < 0.001, ES = 1.74, large), HIRD (p = 0.003, ES = 1.10, moderate), and distance > MAS (p = 0.012, ES = 0.90, moderate), than defenders.

When the team was not in possession of the ball, midfielders, compared to defenders, covered greater distance (p < 0.001, ES = 1.53, large), HSRD (p = 0.006, ES = 1.20, large), HIRD (p = 0.001, ES = 1.32, large), and distance > MAS (p = 0.006, ES = 1.27, large). Compared to forwards, midfielders covered greater total distance (p < 0.001, ES = 1.96, large), HSRD (p < 0.001, ES = 2.09, very large), HIRD (p = 0.001, ES = 2.03, very large), distance > MAS (p = 0.006, ES = 1.93, large), distance > 30% ASR (p < 0.001, ES = 2.14, very large) and distance > 85% PS (p = 0.004, ES = 2.43, very large). Furthermore, defenders covered greater distance > 30% ASR (p = 0.041, ES = 1.25, large), and greater distance > 85% PS (p = 0.047, ES = 1.44, large) than forwards.

During the ball-out-of-play phases, midfielders covered greater total distance compared to defenders (p < 0.001, ES = 1.61, large), and greater distance > MAS (p = 0.005, ES = 2.04, very large) and distance > 30% ASR (p = 0.022, ES = 2.18, very large) compared to forwards. No significant differences were observed between positions for the other variables (all p > 0.05, Table 4).

## DISCUSSION

The study aimed to examine the relationships between different generic and relative speed thresholds in EPL matches across two competitive seasons, and to compare the effect of playing position and possession phase. Taken together, the present findings show low to almost perfect correlations between the examined absolute and relative distances. Distance > 30% ASR was almost perfectly correlated with HSRD, while distances > MAS were highly correlated with both HSRD and HIRD, and distance > 85% PS was highly correlated with sprint distance. During the entire match and when the team was not in possession of the ball, overall, midfielders covered greater distances than forwards and strikers, while forwards covered greater distances than the other two positions when in the ball possession phase. The average values for the generic-threshold distances measured in this study have similar magnitude to those previously reported in studies that assessed physical performance during EPL match-play [46–48].

The analysis of correlations between the examined variables revealed a close-to-perfect correlation between the distance covered > 30% ASR and HSRD. This is unsurprising, as individual 30% MAS in the present sample of players was on average equal to 5.76 ± 0.1 m/s (range 5.43–6.00 m/s), and thus very close to the generic 5.5 m/s threshold for high-speed running and, more importantly, with a low between-player variability (CV = 2.6%). It is worth noting that MAS (4.63 ± 0.19 m/s) and > 85% PS (8.10 ± 0.33 m/s) significant difference for forwards vs. defenders showed slightly higher between-player variability than > 30% ASR (both CV = 4.1%). A possible explanation for this may be the distances based on relative thresholds reporting not perfect correlations, yet still very large to almost perfect, correlations with the respective distances calculated using generic thresholds (rmcorr = 0.91 for distance > MAS vs. HSRD; and rmcorr = 0.70 for distance > 85% PS vs. sprint distance). In the process of load monitoring during training and competition in team sports, it is normally desirable for practitioners to monitor a smaller set of load metrics after reducing the number of available data and select only a few among several correlated variables [49, 50]. From a practical perspective, the present results would seem to suggest that distance covered at > 30% ASR would be unnecessary to assess in elite soccer players during matchplay as long as HSRD is examined, whereas it may still be useful and informative to assess the distance covered > MAS and especially > 85% PS, as these distances, based on relative thresholds, are not perfectly correlated with any generic distance metric. Indeed distance covered > 85% PS may be necessary for practitioners to prepare players for match-play and avoid de-training for non-starters [16]. However, it cannot be excluded that in soccer players of different levels or age categories, or in elite players playing in different elite national leagues, the individual values of > 30% ASR are more variable than in the present metrics, leading to lower relationships with HIRD. In such case, considering distance at speed > 30% ASR may be required to gain detailed information on the actual physical effort of each individual player during match-play.

A further purpose of this study was to compare the physical load across playing positions and possession phases of match-play, such as, TIP, OTIP, and BOP. Indeed, while differences between playing positions for generic distances covered during the entire match have been widely documented in male soccer players at all ages and playing levels [24, 51], only limited knowledge is available regarding positional differences of distances based on MAS, PS, and ASR, or during specific phases related to the ball in/out of play and ball possession. For example, Di Salvo, et al. [46] showed that, in a sample of EPL players competing in the 2003–2004 to 2005–2006 seasons, mean total (whole-match) HSRD (> 5.5 m/s) ranged from 681 m (central defenders) to 1049 m (wide midfielders). Although, the positionspecific HSRD covered ranged from 179 m (central defenders) to 566 m (forwards) when in the ball possession phase, and from 331 m (forwards) to 498 m (wide defenders) in the out-of-possession phase. These ranges reflect the HIRD shown by our sample of players. Furthermore, in the TIP phase, attackers covered the greatest distances among all playing positions, followed by wide midfielders, central midfielders, wide defenders and central defenders. Conversely, in the OTIP phase, the authors showed that almost similar HIRD in all defenders and midfielders, while attackers had a much lower distance covered when compared to other positions. Although the current study classified players into three position categories (defenders, midfielders, forwards) due to examining only one team and thus not having a large enough sample to consider a greater number of categories, our overall results confirm those of Di Salvo, et al. [46] concerning HSRD and extend to other relevant generic and relative distance variables in EPL players.

Indeed, the present study also reported that in the TIP phase, forwards covered greater distances than both defenders and midfielders for most of the examined high-intensity variables, including sprint distance and distance > 85% PS. Whereas for other variables forwards cover lower distances than both midfielders and forwards in the OTIP phase. These findings are likely related to the specific tactical behavior and assigned tasks of players in the TIP and OTIP phases and thus might be slightly different according to a team’s playing formation, to the specific characteristics of individual players (that is, irrespective of their position), and to the specific team defensive and offensive strategies. Instead, our results are only partially consistent with the study of Mendez-Villanueva, et al. [10] where the authors assessed, among other variables, distances > MAS and > 30% ASR during international youth (U13–U19) matches. These authors showed that, especially in the first half of the match, wide midfielders covered greater distances > MAS than full-backs, central backs and central midfielders. Furthermore, central midfielders covered lower distances > 30% ASR than all other positions, and forwards covered a greater distance at such intensity compared to all other positions. The differences between those results and the present findings may be related to the diverse positional definitions utilized and the varying match-play characteristics between youth and elite senior soccer.

Interestingly, in the TIP phase, a significantly greater sprint distance, with large effects in forwards vs. both defenders and midfielders was observed, although these differences were no more evident when considering the distance > 85% PS (Table 3). Similarly, during the OTIP phase, forwards covered significantly less distance > 85% PS than defenders and midfielders, while differences were not significant when considering sprint distance. These discrepancies may be due to the varying individual PS from different playing positions in the present sample, and the > 85% PS (defenders: 8.19 ± 0.30 m/s; midfielders: 7.96 ± 0.36 m/s; forwards: 8.36 ± 0.27 m/s). Thus, considering a relative rather than a generic speed threshold for sprint speed may provide different, and arguably more accurate, information on the actual external load of players due to inter-player and inter-position PS variability.

The distances covered in the BOP phase showed little differences between positions (Table 3). From a practical perspective, distances covered during the BOP phase may not elicit as much information regarding the players’ load and physical performance as TIP and OTIP distances, although still represent a non-negligible quantity of total distance at different intensities and may be interesting from a certain perspective. In agreement with the findings of Mernagh, et al. [52], the current study showed that forwards covered greater distances > MAS and > 30% ASR than midfielders, arguably linked to actions such as re-gaining defensive shape following an attacking phase, to prevent an opponent’s counter-attack concluding with a shot on goal, or, conversely, to occupy an advantageous position prior to the start of a new possession phase.

Despite the previous findings, some limitations of the study should be acknowledge: a) the study was conducted using only one team and thus a limited sample of players were examined, which consequently may restrict a generalization of the results; b) the playing position classification adopted did not allow for the differentiation within each unit; c) the speed distance chosen for this study did not account for the transition between the different speed and intensity zones, usually expressed by accelerometry based variables; d) internal load was not collected which would have strengthened the results of the present study; and e) contextual factors such match location, opponent ranking or match outcome was not considered for analysis that should be encouraged for future studies. Therefore, further research is warranted to assess the distances covered during match-play at relative speed thresholds in players of different levels and of a wider range of MAS, PS and ASR values and examine the nature and practical implications of high-intensity distances covered during ball-out-of-play phases in elite soccer matches. If possible, a greater sample size, acceleration and deceleration data as well as internal load variables should also be included in future studies.

## CONCLUSIONS

In conclusion, the present study showed that, in EPL players, highintensity distances covered during match-play are partially different when calculated using relative, individualized speeds rather than absolute, generic thresholds. This is particularly evident for speed > 85% PS, that showed some relevant differences when compared to speed > 7 m/s. A significant novel finding of this study is that high-intensity distances covered above specific speeds based on individual thresholds have been assessed during official EPL matches, and, in general, in elite soccer players. This provides valuable information for practitioners and researchers on the individual demands of match-play. Although the generic and relative speed thresholds show almost perfect correlation, the differences between HSRD, HIRD and distance > MAS indicate that players may be exposed to more high-intensity distance when using relative thresholds. This knowledge of individualized match demands can aid practitioners in prescribing training loads to athletes. In addition, using thresholds underpinned by scientific rationale may help practitioners reduce injury risk [40] and improve aerobic performance [14] by simply monitoring and adapting to these demands. Future research should aim to examine high-intensity periods as this may highlight greater differences between positions.

## References

[1] Drust B, Atkinson G, Reilly T. Future perspectives in the evaluation of the physiological demands of soccer. Sports Med. 2007; 37(9):783–805. 17722949 10.2165/00007256-200737090-00003

[2] Ravé G, Granacher U, Boullosa D, Hackney AC, Zouhal H. How to Use Global Positioning Systems (GPS) Data to Monitor Training Load in the “Real World” of Elite Soccer. Front Physiol. 2020; 11:944. 32973542 10.3389/fphys.2020.00944 PMC7468376

[3] Morgans R, Rhodes D, Teixeira J, Modric T, Versic S, Oliveira R. Quantification of training load across two competitive seasons in elite senior and youth male soccer players from an English Premiership club. Biol Sport. 2023; 40(4):1197–1205. 37867738 10.5114/biolsport.2023.126667 PMC10588577

[4] Akenhead R, Hayes PR, Thompson KG, French D. Diminutions of acceleration and deceleration output during professional football match play. J Sci Med Sport. 2013 Nov; 16(6):556–61. doi: 10.1016/j.jsams.2012.12.005. 23333009

[5] Hunter F, Bray J, Towlson C, et al. Individualisation of time-motion analysis: a method comparison and case report series. *Int J Sports Med*. 2015; 36(1):41–48. doi:10.1055/s-0034-1384547. 25259591

[6] Miguel M, Oliveira R, Loureiro N, García-Rubio J, Ibáñez SJ. Load measures in training/match monitoring in soccer: A systematic review. Int J Environ Res Public Health. 2021; 18(5):2721. 33800275 10.3390/ijerph18052721 PMC7967450

[7] Rico-González M, Oliveira R, Vieira LHP, Pino-Ortega J, Clemente F. Players’ performance during worst-case scenarios in professional soccer matches: a systematic review. Biol Sport. 2022; 39(3):695–713. 35959320 10.5114/biolsport.2022.107022 PMC9331336

[8] Abbott W, Brickley G, Smeeton NJ. An individual approach to monitoring locomotive training load in English Premier League academy soccer players. Int J Sports Sci Coach. 2018; 13(3):421–428. doi: 10.1177/1747954118771181.

[9] Abt G, Lovell R. The use of individualized speed and intensity thresholds for determining the distance run at high-intensity in professional soccer. J Sports Sci. 2009 Jul; 27(9):893–8. doi: 10.1080/02640410902998239. 19629838

[10] Mendez-Villanueva A, Buchheit M, Simpson B, Bourdon PC. Match play intensity distribution in youth soccer. Int J Sports Med. 2013 Feb; 34(2):101–10. doi: 10.1055/s-0032-1306323. 22960988 10.1055/s-0032-1306323

[11] Sonderegger K, Tschopp M, Taube W. The challenge of evaluating the intensity of short actions in soccer: a new methodological approach using percentage acceleration. PloS One. 2016; 11(11):e0166534. 27846308 10.1371/journal.pone.0166534 PMC5112910

[12] Baker D, Heaney N. Review of the literature normative data for maximal aerobic speed for field sport athletes: a brief review. J Aust Strength Cond. 2015; 23(7):60–67.

[13] Gabbett TJ. Use of Relative Speed Zones Increases the High-Speed Running Performed in Team Sport Match Play. J Strength Cond Res. 2015 Dec; 29(12):3353–9. doi: 10.1519/JSC.0000000000001016. 26020710

[14] Fitzpatrick JF, Hicks KM, Hayes PR. Dose-Response Relationship Between Training Load and Changes in Aerobic Fitness in Professional Youth Soccer Players. Int J Sports Physiol Perform. 2018 Nov 19; 1–6. doi: 10.1123/ijspp.2017-0843. 29745785

[15] Kavanagh R, Carling C, Malone S, Di Michele R, Morgans R, Rhodes D. ‘Bridging the gap’: Differences in training and match physical load in 1^st^ team and U23 players from the English Premier League. 2023.

[16] Gualtieri A, Rampinini E, Sassi R, Beato M. Workload monitoring in top-level soccer players during congested fixture periods. Int J Sports Med. 2020; 41(10):677–681. 32455455 10.1055/a-1171-1865

[17] Padrón-Cabo A, Solleiro-Duran D, Lorenzo-Martínez M, Nakamura FY, Campos-Vázquez M, Rey E. Application of arbitrary and individualized load quantification strategies over the weekly microcycle in professional soccer players. Biol Sport. 2023; 41(1):153–161. 38188102 10.5114/biolsport.2024.129481 PMC10765452

[18] Clemente FM, Ramirez-Campillo R, Beato M, Moran J, Kawczynski A, Makar P, Sarmento H, Afonso J. Arbitrary absolute vs. individualized running speed thresholds in team sports: A scoping review with evidence gap map. Biol Sport. 2023; 40(3):919–943. doi: 10.5114/biolsport.2023.122480. 37398971 PMC10286616

[19] Akenhead R, Nassis GP. Training Load and Player Monitoring in High-Level Football: Current Practice and Perceptions. Int J Sports Physiol Perform. 2016 Jul; 11(5):587–93. doi: 10.1123/ijspp.2015-0331. 26456711

[20] Morgans R, Orme P, Bezuglov E, Di Michele R. Technical and physical performance across five consecutive seasons in elite European Soccer. Int J Sports Sci Coach. 2022.

[21] Abbott W, Brickley G, Smeeton NJ. Physical demands of playing position within English Premier League academy soccer. J Hum Sport Exerc. 2018; 13(2).

[22] Bloomfield J, Polman R, O’Donoghue P. Physical demands of different positions in FA Premier League soccer. J Sports Sci Med. 2007; 6(1):63. 24149226 PMC3778701

[23] Bradley PS, Lago-Peñas C, Rey E, Gomez Diaz A. The effect of high and low percentage ball possession on physical and technical profiles in English FA Premier League soccer matches. J Sports Sci. 2013; 31(12):1261–1270. 23697463 10.1080/02640414.2013.786185

[24] Lorenzo-Martinez M, Kalen A, Rey E, Lopez-Del Campo R, Resta R, Lago-Penas C. Do elite soccer players cover less distance when their team spent more time in possession of the ball? Sci Med Footb. 2021 Nov; 5(4):310–316. doi: 10.1080/24733938.2020.1853211. 35077300

[25] Morgans R, Kweon D, Ryan B, et al. Playing position and match location affect the number of high-intensity efforts more than the quality of the opposition in elite football players. Biol Sport. 2024; 41(3):29–37. doi:10.5114/biolsport.2024.133669. PMC11167469 38952904

[26] Chmura P, Konefał M, Chmura J, Kowalczuk E, Zając T, Rokita A, Andrzejewski M. Match outcome and running performance in different intensity ranges among elite soccer players. Biol Sport. 2018; 35(2):197–203. doi: 10.5114/biolsport.2018.74196. 30455549 PMC6234309

[27] Faude O, Koch T, Meyer T. Straight sprinting is the most frequent action in goal situations in professional football. J Sports Sci. 2012; 30(7):625–631. 22394328 10.1080/02640414.2012.665940

[28] Modric T, Versic S, Morgans R, Sekulic D. Match running performance characterizing the most elite soccer match-play. Biol Sport. 2023; 40(4):949–958. 37867756 10.5114/biolsport.2023.124847 PMC10588580

[29] Aalbers B, Van Haaren J. Distinguishing between roles of football players in play-by-play match event data. In: Machine Learning and Data Mining for Sports Analytics: 5th International Workshop, MLSA 2018, Co-located with ECML/PKDD 2018, Dublin, Ireland, September 10, 2018, Proceedings 5. Springer; 2019:31–41.

[30] Trewin J, Meylan C, Varley MC, Cronin J. The influence of situational and environmental factors on match-running in soccer: a systematic review. Sci Med Football. 2017; 1(2):183–194.

[31] Oliveira R, Brito J, Martins A, Mendes B, Calvete F, Carriço S, Ferraz R, Marques MC. In-season training load quantification of one-, two- and three-game week schedules in a top European professional soccer team. Physiol Behav. 2019 Mar 15; 201:146–156. doi: 10.1016/j.physbeh.2018.11.036. 30529511

[32] Nobari H, Brito JP, Pérez-Gómez J, Oliveira R. Variability of external intensity comparisons between official and friendly soccer matches in professional male players. Healthcare. 2021; 9(12):1708. 34946434 10.3390/healthcare9121708 PMC8702108

[33] Nobari H, Khalili SM, Oliveira R, Castillo-Rodríguez A, Pérez-Gómez J, Ardigò LP. Comparison of official and friendly matches through acceleration, deceleration and metabolic power measures: a full-season study in professional soccer players. Int J Environ Res Public Health. 2021; 18(11):5980. 34199573 10.3390/ijerph18115980 PMC8199659

[34] Ingebrigtsen J, Dalen T, Hjelde GH, Drust B, Wisløff U. Acceleration and sprint profiles of a professional elite football team in match play. Eur J Sport Sci. 2015; 15(2):101–110. 25005777 10.1080/17461391.2014.933879

[35] Bradley PS, Sheldon W, Wooster B, Olsen P, Boanas P, Krustrup P. High-intensity running in English FA Premier League soccer matches. J Sports Sci. 2009; 27(2):159–168. 19153866 10.1080/02640410802512775

[36] Kavanagh R, McDaid K, Rhodes D, McDonnell J, Oliveira R, Morgans R. An Analysis of Positional Generic and Individualized Speed Thresholds Within the Most Demanding Phases of Match Play in the English Premier League. Int J Sports Physiol Perform. 2023; 1–11. 10.1123/ijspp.2023-0063 38134895

[37] Winter EM, Maughan RJ. Requirements for ethics approvals. 2009. 10.1080/02640410903178344 19847681

[38] Bezuglov E, Talibov O, Khaitin V, Pirmakhanov B, Waśkiewicz Z, Butovskiy M, Morgans R. Running Performance during the Holy Month of Ramadan in Elite Professional Adult Soccer Players in Russia. Int J Environ Res Public Health. 2021 Nov 8; 18(21):11731. doi: 10.3390/ijerph182111731. PMID:34770245; PMCID: . 34770245 PMC8583445

[39] FIFA. Test Report. 17-11-2022. 2021. [Online]. Available: https://www.fifa.com/technical/football-technology/resource-hub?id=440b5a4c9b08464898bf51a08c0b8a5e.

[40] Colby MJ, et al. Improvement of Prediction of Noncontact Injury in Elite Australian Footballers With Repeated Exposure to Established High-Risk Workload Scenarios. Int J Sports Physiol Perform. 2018 Oct 1; 13(9):1130–1135. doi: 10.1123/ijspp.2017-0696. 29543079

[41] Kelly V, Wood A. The correlation between the 30–15 intermittent fitness test and a novel test of running performance. J Aust Strength Cond. 2013; 21(S1):91–94.

[42] Berthon P, Fellmann N, Bedu M, Beaune B, Dabonneville M, Coudert J, Chamoux A. A 5-min running field test as a measurement of maximal aerobic velocity. Eur J Appl Physiol Occup Physiol. 1997; 75(3):233–8. doi: 10.1007/s004210050153. PMID:9088842. 9088842

[43] Knicker AJ, Renshaw I, Oldham AR, Cairns SP. Interactive processes link the multiple symptoms of fatigue in sport competition. Sports med. 2011; 41(4):307–328. 21425889 10.2165/11586070-000000000-00000

[44] Bakdash JZ, Marusich LR. Repeated measures correlation. Front Psychol. 2017; 8:456. 28439244 10.3389/fpsyg.2017.00456 PMC5383908

[45] Hopkins W, Marshall S, Batterham A, Hanin J. Progressive statistics for studies in sports medicine and exercise science. Med Sci Sports Exerc. 2009; 41(1):3. 19092709 10.1249/MSS.0b013e31818cb278

[46] Di Salvo V, Gregson W, Atkinson G, Tordoff P, Drust B. Analysis of high intensity activity in Premier League soccer. Int J Sports Med. 2009; 30(03):205–212. 19214939 10.1055/s-0028-1105950

[47] Barnes C, Archer D, Hogg B, Bush M, Bradley P. The evolution of physical and technical performance parameters in the English Premier League. Int J Sports Med. 2014; 35(13):1095–1100. 25009969 10.1055/s-0034-1375695

[48] Bush M, Barnes C, Archer DT, Hogg B, Bradley PS. Evolution of match performance parameters for various playing positions in the English Premier League. Hum Mov Sci. 2015; 39:1–11. 25461429 10.1016/j.humov.2014.10.003

[49] Pexa B, Ryan ED, Blackburn JT, Padua DA, Garrison JC, Myers JB. Influence of baseball training load on clinical reach tests and grip strength in collegiate baseball players. J Athl Train. 2020; 55(9):984–993. 32857132 10.4085/1062-6050-0456.19 PMC7534943

[50] Williams S, Trewartha G, Cross MJ, Kemp S, Stokes KA. Monitoring What Matters: A Systematic Process for Selecting Training-Load Measures. Int J Sports Physiol Perform. 2017; 12. 10.1123/ijspp.2016-0337 27834553

[51] da Mota GR, Thiengo CR, Gimenes SV, Bradley PS. The effects of ball possession status on physical and technical indicators during the 2014 FIFA World Cup Finals. J Sports Sci. 2016; 34(6):493–500. 26703781 10.1080/02640414.2015.1114660

[52] Mernagh D, Weldon A, Wass J, et al. A Comparison of Match Demands Using Ball-in-Play versus Whole Match Data in Professional Soccer Players of the English Championship. Sports (Basel). 2021; 9(6):76. Published 2021 May 26. doi:10.3390/sports9060076. 34073473 PMC8228731

